# Potential Biomarkers in Mouse Myocardium of Doxorubicin-Induced Cardiomyopathy: A Metabonomic Method and Its Application

**DOI:** 10.1371/journal.pone.0027683

**Published:** 2011-11-16

**Authors:** Guangguo Tan, Ziyang Lou, Wenting Liao, Zhenyu Zhu, Xin Dong, Wei Zhang, Wuhong Li, Yifeng Chai

**Affiliations:** 1 School of Pharmacy, Second Military Medical University, Shanghai, China; 2 Shanghai Key Laboratory for Pharmaceutical Metabolite Research, Shanghai, China; Roswell Park Cancer Institute, United States of America

## Abstract

**Background:**

Doxorubicin (DOX) is one of the most potent antitumor agents available; however, its clinical use is limited because of the risk of severe cardiotoxicity. Though numerous studies have ascribed DOX cardiomyopathy to specific cellular pathways, the precise mechanism remains obscure. *Sini* decoction (*SND*) is a well-known formula of Traditional Chinese Medicine (TCM) and is considered as efficient agents against DOX-induced cardiomyopathy. However, its action mechanisms are not well known due to its complex components.

**Methodology/Principal Findings:**

A tissue-targeted metabonomic method using gas chromatography–mass spectrometry was developed to characterize the metabolic profile of DOX-induced cardiomyopathy in mice. With Elastic Net for classification and selection of biomarkers, twenty-four metabolites corresponding to DOX-induced cardiomyopathy were screened out, primarily involving glycolysis, lipid metabolism, citrate cycle, and some amino acids metabolism. With these altered metabolic pathways as possible drug targets, we systematically analyzed the protective effect of TCM *SND*, which showed that *SND* administration could provide satisfactory effect on DOX-induced cardiomyopathy through partially regulating the perturbed metabolic pathways.

**Conclusions/Significance:**

The results of the present study not only gave rise to a systematic view of the development of DOX-induced cardiomyopathy but also provided the theoretical basis to prevent or modify expected damage.

## Introduction

Doxorubicin (DOX) is a powerful anticancer antibiotic [1]. The clinical use of DOX is often limited because of its undesirable serious cardiotoxic side effects, which frequently lead to congestive heart failure [2], [3], [4]. Though numerous studies have ascribed doxorubicin cardiotoxicity to specific cellular pathways, including an increase in free radicals formation [5], lipid peroxidation [6], cardiomyocyte apoptosis [7], [8], interference of calcium dynamics [9], abnormalities in the mitochondria [10], [11], [12], alteration of cardiac energetics [13], [14], [15], irreversible damage of DNA [16] and other putative mechanisms, the precise mechanism of this pathogenesis remains obscure. Therefore the biological processes of doxorubicin-induced cardiomyopathy still need further investigation to find its biomarkers and illuminate its mechanism.

Medicinal plants play a key role in human health care nowadays. It is increasingly being understood that the Traditional Chinese Medicine (TCM) does have a remedy for acute or chronic disorders. *Sini* decoction (*SND*), which is officially recorded in the Chinese Pharmacopoeia 2010 edition and has been used to prevent or treat cardiovascular disease as well as DOX-induced cardiomyopathy for many years [17], [18], [19], [20], is a representative TCM. It is composed of three medicinal plants: *Acontium carmichaeli*, *Glycyrrhiza uralensis* and *Zingiber officinale*. As there are complex components in *SND*, which can hit multiple targets and exert an overall therapeutic effect, it is hard to understand its action mechanisms completely.

Metabonomics, the systems biology approach of small molecules, is a rapidly advancing field that aims to monitor as many metabolites with low molecular weight as possible rather than focus on individual metabolite in a single cell, biofluids, and tissue extracts [21], [22]. It provides insights into the global metabolic state of entire organism, which is well coincident with the integrity and systemic feature of TCM [23], [24]. The emerging metabonomics has been conducted to analyze doxorubicin related toxicity biomarkers [25], [26]. For example, J. C. Park [25] identified 19 urinary metabolites in rats treated with doxorubicin. Those identified biomarkers suggested renal dysfunction and liver injury as well as impairment of energy metabolism. The study of J.S. Wang [26] showed that tryptophan and phenylalanine metabolism was also important process in the systemic toxicity of doxorubicin.

Though a lot of urinary biomarkers have been successfully identified from the systemic toxicity of doxorubicin, the tissue-specific biomarkers in myocardium still need further study to provide a new insight into the pathological processes of doxorubicin-induced cardiomyopathy. Therefore, a gas chromatography–mass spectrometry (GC-MS) method based on a metabonomic strategy was developed. In addition, the reverse effects of TCM *SND* and its mechanisms were also investigated by using a metabonomic approach for the first time.

## Results

### Serum biochemical assay

**Figure 1:**
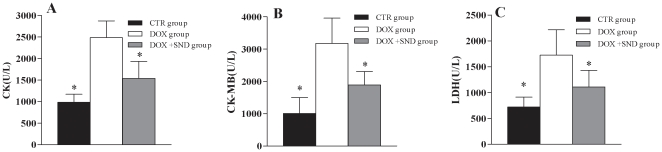
CK, CK-MB, and LDH were significantly elevated in DOX treated rats compared to the control group and to the *SND* treated groups. (A) Serum creatine phosphokinase (CK). (B) Serum creatine phosphokinase-MB (CK-MB) and (C) lactate dehydrogenase (LDH). Data are expressed as mean ± S.D. *p<0.05 versus DOX group.

Serum levels of creatine kinases (CK), creatine kinase-MB (CK-MB) and lactate dehydrogenase (LDH) were significantly elevated 72 h after DOX injection compared with the levels in the control rats (**Figure 1A–C**). *SND* significantly attenuated DOX-induced increase in the above enzymes in serum, indicative of reduced myocardial necrosis (*p*<0.05).

### Selection of DOX-induced metabolites

**Figure 2:**
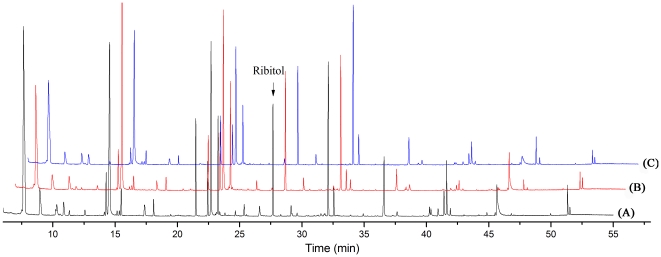
The typical total ions current chromatograms (TICs) of myocardial extract. (A) control group; (B) DOX group; (C) DOX+*SND* group.

**Figure 3:**
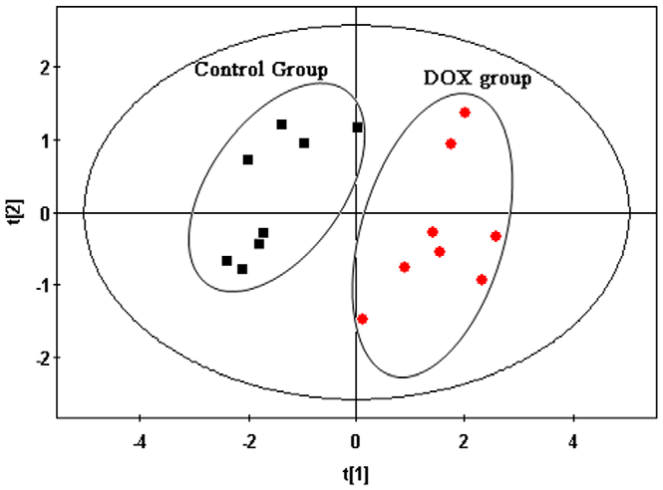
PCA score plot derived from myocardial extracts GC-MS spectra concerning control (square) and DOX (circle) groups.

**Figure 4:**
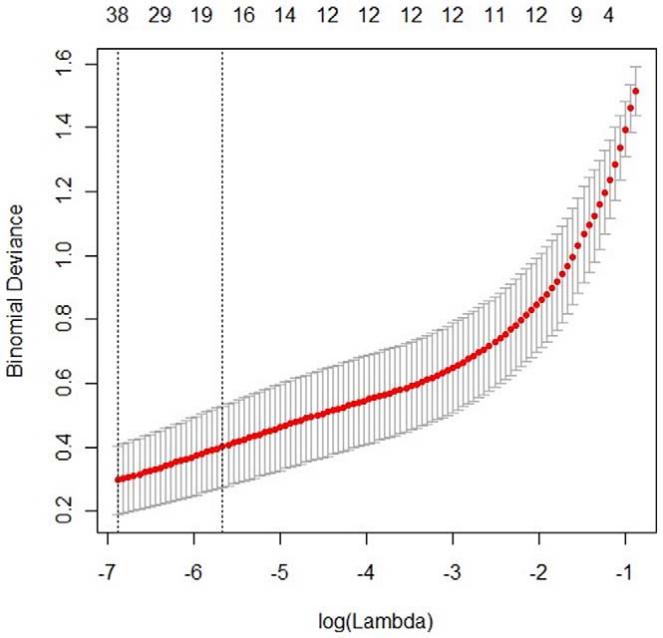
The relationship between lambda and deviance using the Elastic Net approach.

The typical total ion current chromatograms (TICs) of myocardial extracts are shown in **Figure 2**. After aligning mass ions using XCMS software, 1371 ions peaks were obtained, where a few of these fragment ion peaks were found from the same silylation derivatives of endogenous metabolites. By untargeted filtration of ion peaks (It was described in the **Material and Methods** section), the data was simplified and 170 ion peaks were obtained. The unsupervised Principal Component Analysis (PCA) was first applied to explore correlations between control and DOX groups, and a tendency in the score plot to separate the two classes was detected (*R* ^2^ = 0.50) (**Figure 3**). To further search ion peaks that can discriminate between groups, a unique challenge is posed because of a large number of variables, and a few observations. The traditional partial least squares (PLS) method has extensively applied to selection of biomarkers in the similar metabonomic data [27]. Unfortunately, it cannot automatically retain good features. In this study, a recent developed approach, Elastic Net, was used for classification and selection of biomarkers [28]. An advantage of the Elastic Net approach is that some of the effects of the variables in these models are estimated to be exactly zero. These will represent variables that have no discriminatory power between the two classes, while those with nonzero coefficients will represent variables that can separate classes of groups successfully. Thus, a variable list that can successfully discriminate the classes could be automatically obtained. **Figure 4** shows the relationship between lambda and deviance using the Elastic Net approach for the 170 ion peaks with 5-fold cross-validation. We can found that there was a small deviance for classification when we selected specific lambda value, an important parameter in Elastic Net approach, which can produce a classifier with 37 variables plus an intercept term. Thus, 37 variables with nonzero coefficients were first selected as the candidates of potential biomarkers. Some of these variables were found to be from the same metabolites. After merging the variables from the identical metabolites, 24 metabolites were collected and considered as the potential biomarkers. To accurately evaluate their changes, the ratios of their actual peak areas to that of the internal standard in both control group and DOX group were calculated and then validated using one-way analysis of variance (ANOVA) (*p*<0.05). Finally, it was found that sixteen of 24 metabolites, including L-alanine, phosphate, glycine, succinate, malate, proline, threonic acid, glutamine, phenylalanine, dihydroxyacetonephosphate (DHAP), glycerol-3-phosphate (G-3-P), fructose, glucose, stearic acid, myo-inositol and cholesterol, were evaluated in the DOX group, while the other eight metabolites including lactate, β-hydroxybutyric acid, L-valine, isoleucine, threonine, citrate, linoleic acid, arachidonic acid, were reduced in the DOX group. (Table S1).

### Effect of *SND* administration

**Figure 5:**
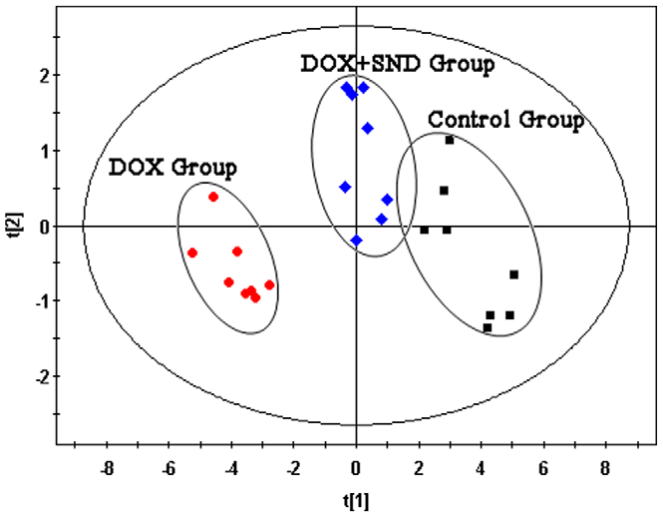
PCA scores plot derived from myocardial levels of twenty-four metabolites in control (square), DOX (circle) and DOX plus *SND* (diamond) groups.

**Figure 6:**
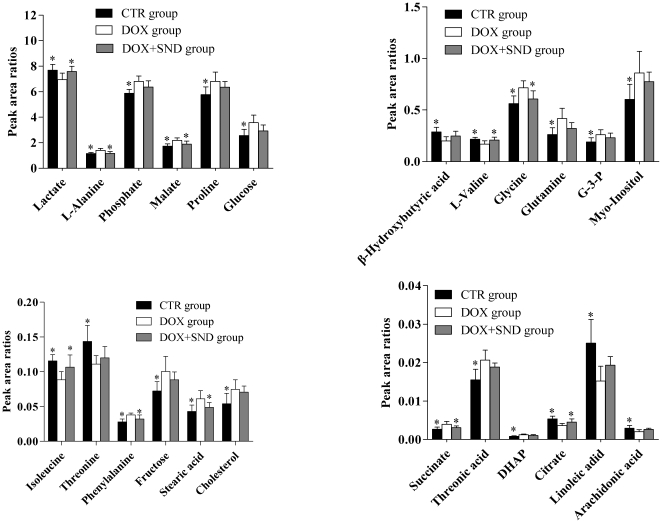
Bar graphs show the relative peak area ratios of the 24 metabolites to ribitol in CTR, DOX, and DOX plus *SND* groups. Data are expressed as mean ± S.D.. *p<0.05 versus DOX group.

The effective results of *SND* for protection of DOX-induced cardiomyopathy have been shown in Section “**Serum biochemical assay**”. As the 24 potential biomarkers have been found, it is reasonable to take them as the monitoring indexes for further investigating the protective mechanisms of *SND* to DOX-induced cardiomyopathy. Therefore, the levels of these 24 biomarkers were introduced as variables to PCA, performed on three groups. As shown in **Figure 5**, the DOX plus *SND* group was closer to the control group, which might suggest *SND* can reverse the pathological process of DOX-induced cardiomyopathy. To further evaluate the reversed condition of the potential biomarkers by pre-administration with *SND*, one-way ANOVA with Tukey post hoc test was performed by SPSS software. The ratios of relative peak area of the 24 metabolites to that of ribitol are shown in **Figure 6**. Compared to the DOX group, the levels of ten metabolites including lactate, L-alanine, L-valine, isoleucine, glycine, succinate, malate, phenylalanine, citrate, and stearic acid were significantly reversed in DOX plus *SND* group. The other fourteen metabolites were also reversed at different degrees.

## Discussion

The present study demonstrates, for the first time, a GC-MS-based metabonomic analysis of DOX-induced cardiomyopathy in mice, identifying 24 potential biomarkers relevant to the disturbance of myocardial energy metabolism primarily involving glycolysis, lipid metabolism, citrate cycle, and some amino acids metabolism. Furthermore, it was confirmed that Chinese Medicine *SND* could play its role in DOX-induced cardiomyopathy by intervening in some metabolic pathways, such as glycolysis, lipid metabolism, citrate cycle, and some amino acids metabolism, which might be mapped by restoring alterations in some biomarkers found in this research.

Among these metabolites, lactate, fructose, glucose, DHAP and G-3-P are correlated with glycolysis, indicating the modulation of the glycolytic pathway. A reduced in lactate level was observed in DOX-induced group. It is conceivable that the reduction should be accompanied with the regulation of enzymes in the heart. The lactate dehydrogenase B (LDHB), a subtype of lactate dehydrogenase mainly existing in myocardium, was recently found to be up-regulated in the DOX treated cardiomyocytes [29], which could result in lactate reduction through the conversion of lactate to pyruvate. The elevated glucose and fructose in DOX-induced group could result from the decreased utilization of glucose and other simple sugars, which was compatible with the previous findings [15], [30], [31]. The build-up of DHAP together with G-3-P was also observed in DOX-induced group, which was agreed with the results of Ronen *et al.* [32]. A more likely explanation for the elevation is inhibition of the flux through the glycolytic pathway at the level of glyceraldehyde-3-phosphate dehydrogenase (GAPDH). GAPDH catalyses the nicotinamide adenine dinucleotidedependent conversion of glyceraldehyde-3-phosphate (GADP) into 1,3-diphosphoglycerate (1, 3-DPG). It is the first energy-harvesting enzyme and a central player in glycolytic pathway. The previous study confirmed the supposition, where the lower expression of GAPDH was observed in DOX-induced bovine pulmonary artery endothelial cells [33].

The citrate cycle is central to aerobic metabolism, facilitating adequate throughout of substrates derived from carbohydrates, fatty acids or certain amino acids [34]. Three pivotal intermediates of citrate cycle were changed in this study, where the low level of citrate and high level of succinate and malate were observed in DOX-induced group. It has been reported that DOX-induced cardiomyopathy is associated with a decreased utilization of both substrates, fatty acids and glucose [15], [35], which could, at least in part, lead to the reduction of citrate synthesis. However, the elevation of succinate and malate could result from the regulation of some key enzymes associated with the citrate cycle. As we know, the metabolism of succinate and malate depend on succinate dehydrogenase (SDH) and malate dehydrogenase (MDH). We speculated that the decrease in activities of SDH and MDH should be a reason for the accumulation of succinate and malate, respectively. The previous study by Gnanapragasam *et al.* supported that the activities of SDH and MDH indeed decreased in DOX-treated rats [36].

Lipid metabolism has a key role in DOX-induced cardiomyopathy, which has been demonstrated by numerous studies [37], [38], [39]. Lipid-based metabolites play an important role in many biochemistry reactions and are related to many biological functions. In this study, the levels of one saturated fatty acid (stearic acid) and two unsaturated fatty acids (arachidonic acid and linoleic acid) were significantly changed. An interesting finding is that their changes were quite different, where the saturated fatty acid was upgraded while the unsaturated fatty acids were downgraded. The elevated stearic acid in DOX treated mice could indicate that the β-oxidation of the saturated fatty acid was inhibited. It is known that β-oxidation of fatty acids is tightly associated with glycometabolism in the heart. The inhibition of citrate cycle and oxidative phosphorylation due to DOX accumulation in the heart should be the main cause of fatty acids oxidation attenuating. Therefore, the accumulation of acyl-CoA and NADH should have occurred in DOX-induced cardiocytes. The down-regulating of two enzymes, carnitine palmitoyltransferase I (CPTI) and NADH dehydrogenase (NADHD) in DOX-induced cardiocytes were found by the previous studies [39], [40], which provides the indirect evidence to the speculation. However, arachidonic acid and linoleic acid, two unsaturated fatty acids, were present in decreased level in DOX-induced group. One possible explanation was that DOX caused the peroxidation of unsaturated fatty acids [6]. The abnormal oxidation status contributed to excessive oxidation damage on myocardial mitochondrial. Several studies have reported that the level of malondialdehyde (MDA), the end product of these unsaturated fatty acids peroxidation, was increased in DOX-treated mice, which also indirectly confirmed the supposition [36], [41]. In addition, cholesterol was elevated in DOX-induced group. Previous studies have been proved that DOX could reduce the rate of lipolysis [37], suggesting that the accumulation of cholesterol was a characteristic of DOX-induced cardiotoxicity.

The low levels of valine and isoleucine, two branched-chain amino acids (BCAAs), were observed in DOX group. BCAAs may be an important alternative energy substrate for the heart in myocardial ischemia [42]. It seems that the reduction of ATP production, due to the inhibition of β-oxidation of fatty acids and citrate cycle induced by DOX, could lead to the utilization of BCAAs as energy compensation. GC-MS spectra also showed the changes of other α-amino acids metabolism, where a decrease in threonine and a build-up in L-alanine, gylcine, phenylalanine, L-proline and glutamine were observed in DOX group. α-amino acids are important energy metabolism precursors and can be transformed into some biomolecules, such as pyruvate, 2-oxoglutarate and fumarate, to enter into citrate cycle. One possible speculation was that oxidative stress caused by DOX lead to the metabolic remodeling of α-amino acids to meet energy requirement in myocardium [43]. In addition, the alterations in myocardial energy metabolism induced by DOX were associated with ATP depletion and the accumulation of phosphate [44]. Finally, the level of β-hydroxybutyric acid, threonine and myo-inositol was also changed in myocardial tissue, which was perplexing corresponding to DOX induction due to lack of information about their biology pathways.

**Figure 7:**
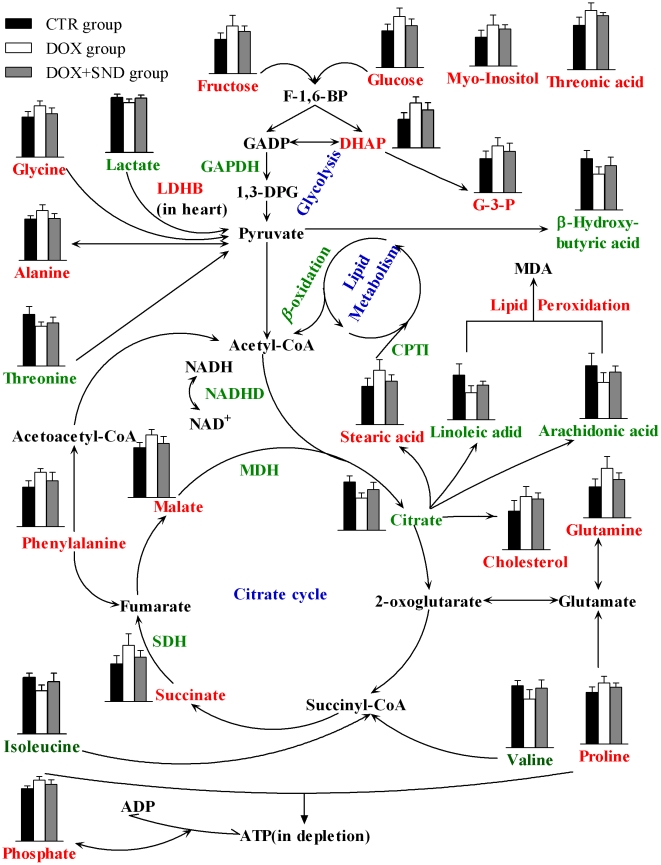
Schematic overview of the metabolites changing for doxorubicin cardiomyopathy in myocardium and *SND* modulation. Column value in histograms is expressed as mean ± S.D.. Metabolites or proteins in red and green represent elevation and inhibition, respectively. The expression of the proteins was based on the previous studies. Metabolites in black means they were not detected in our experiment. The blue words are pathway's names.

It was reported that *SND* could up-regulate B-cell lymphoma-extra large (Bcl-xl) protein and down-regulate BH3 interacting domain death agonist protein in DOX-induced cardiomyocytes [17], resulting in promoting mitochondrial function and reducing apoptosis, which might be responsible for the protective effect of *SND*. In this study, the down-regulation of succinate, malate, and stearic acid and up-regulation of lactate and citrate were observed in DOX plus *SND* group compared with DOX group, which implied that *SND* might functionally intervene in glycolysis, lipid metabolism and citrate cycle. In fact, *SND* administration permitted the mean levels of each potential biomarker to reverse at different degrees. These results combined with the serum biomedical assay suggested that *SND* has unique characteristics for the protective effect on DOX-induced cardiomyopathy. The potential biomarkers revealed metabolic pathways (glycolysis, lipid metabolism, citrate cycle, and amino acids metabolism) might be involved in the protective mechanism of *SND*. Based on these discussions, the schematic overview of the potential biomarkers changing for DOX-induced cardiomyopathy in cardiac muscle and *SND* modulation is shown in **Figure 7**, which is helpful to understand the development of DOX cardiomyopathy and mode-of-action of *SND*.

In conclusion, a metabonomic study of the biochemical profiles of DOX-induced cardiomyopathy was performed using GC-MS and multivariate analysis. Such a study allowed the holistic evaluation of changes in the level of metabolites. With Elastic Net for classification and selection of biomarkers, twenty-four metabolites primarily involved in glycolysis, citrate cycle, lipid metabolism and amino acids metabolism, were screened out and considered as potential biomarkers corresponding to DOX-induced cardiomyopathy. Combined with the previous studies, the metabolites' change shed new light on DOX cardiomyopathy, which would be used to understand the development of the disease and assess drug efficacy as well as and mode-of-action. The GC-MS-based metabonomic method has been successfully applied to evaluate the protective effect of TCM *SND*. Taking the potential biomarkers found in the study as screening indexes, it revealed that *SND* could restore the unbalanced glycolysis, lipid metabolism, citrate cycle, and amino acids metabolism. Therefore, it is suggested that TCM *SND* was a promising potential cardioprotection agent against DOX-induced toxicity.

## Materials and Methods

### Ethics Statement

All animal experiments were approved by the Administrative Committee of Experimental Animal Care and Use of Second Military Medical University (SCXK(Hu)2007-0005), and conformed to the National Institute of Health guidelines on the ethical use of animals.

### Reagents and materials

The assay kits for creatine kinases (CK), creatine kinase-MB (CK-MB) and lactate dehydrogenase (LDH) were purchased from Shanghai Fosun Long March Medical Science Co. Methoxylamine hydrochloride, *N*-methyl-*N*-(trimethylsilyl)- trifluoracetamide (MSTFA), pyridine, trimethyl-chlorosilane (TMCS), *n*-heptane, acetone, β-hydroxybutyric acid and citrate were purchased from Sigma-Aldrich (St Louis, MO, USA). Methanol and acetonitrile are chromatography pure (Merk, Germany). The following compounds were obtained from Shanghai Jingchun Reagent Co.: ribitol, lactate, L-alanine, L-valine, phosphate, isoleucine, glycine, Sodium succinate, threonine, malate, L-proline, L-threonic acid hemicalcium salt, L-glutamine, phenylalanine, fructose, glucose, myo-inositol, linoleic acid, stearic acid, arachidonic acid and cholesterol.

*Acontium carmichaeli* (collection in Sichuan, China), *Glycyrrhiza uralensis* (collection in Xinjiang, China) and *Zingiber officinale* (collection in Guizhuo, China) were purchased from Shanghai Dekang Medicine Corp. (Shanghai, China) were authenticated by *Lianna Sun* (Department of Pharmacognosy, School of Pharmacy, Second Military Medical University, Shanghai, China).

### Preparation of *SND* and phytochemical investigation

According to the original composition of *SND* recorded in Chinese Pharmacopoeia 2010 edition, it was prepared using the following procedure. The crude drugs of *A. carmichaeli* 60 g, *G. uralensis* 60 g and *Z. officinale* 40 g were immersed in 1.6 liter water for 1 h and then decocted to boil for 2 h. The decoction was filtered through four layers of gauze. Next, the dregs were boiled once again for 1 h with 1.2 liters of water and the decoction was filtrated out with the above method. Afterward, the successive decoctions were merged and condensed by a rotatory evaporator under reduced pressure. Finally, the extraction solution was made to a concentration of 1.0 g/mL (expressed as the weight of raw materials). According to our previous published paper [45], 53 components of *SND* were identified.

### Animal experiments

All animal experiments were performed at the Centre of Laboratory Animals of the Second Military Medical University (Shanghai, China) in accordance with the relevant national legislation and local guidelines. ICR mice, 25∼30 g weight, were purchased from the Shanghai Laboratory Animal Co. Mice were housed with temperature range from 20°C to 25°C and humidity range from 50% to 60%. The mice were fed with commercial aseptic food and tap water *ad libitum* throughout the experimental period. The animals were randomly divided into three groups (n = 8). Control group: 200 µL of normal saline were injected i.p. DOX group: animals of the DOX group received a single dose of DOX (20 mg/kg, i.p.). DOX plus *SND* group: the mice were treated with *SND* 10 g/kg/BW o.p. for 7 consecutive days as follows: 4 days before, on the same day, and 2 days after administration of a single dose of DOX (20 mg/kg, i.p.). The administration dose in the current study is in accordance with clinical use. Seventy-two hours after DOX administration, blood samples were collected for biomedical measurement including CK, CK-MB and LDH and hearts were rapidly excised and frozen in liquid nitrogen for metabonomic analysis.

### Sample preparation

Myocardial tissue (∼50 mg) was homogenized in 200 µL saline and then centrifuged at 13,000×g for 15 min at 4°C. 100-µL aliquot of the supernatant was transferred into a centrifuge tube and spiked with the internal standard (5 µL of 1 mg/ml of ribitol), followed by adding 400 µL ice cold methanol ∶ acetonitrile ∶ acetone (1∶1∶1) into the tube. After vigorous shaking for 30 s and incubation on ice for 10 min, the mixture was centrifuged at 14,000×g for 15 min to precipitate the protein. The supernatant (300 µL) was transferred into a GC vial and evaporated to dryness under N_2_ stream at room temperature. The derivatization was performed using methoxyamine pyridine (75 µL; 15 mg/mL) at 70°C for 1 h, followed by MSTFA (75 µL) with 1% TMCS as catalyst at room temperature for 1 h. *n*-Heptane (200 µL) was added to dilute the solution after the derivatization and the supernatant was used for GC-MS analysis.

### GC-MS analysis

The derivatized samples for GC-MS were analyzed on a Thermo-Finnigan Trace DSQ fasting scanning single-quadrupole MS (Thermo Electron Corporation) operated at unit mass resolving power. A 1.0 µL of sample solution was injected with splitless mode to TB-5MS column (30 m×250 µm×0.25 µm) with helium as the carrier gas at a flow of 1.0 mL/min. The injector temperature was set at 260°C. The temperature of the ion source was adjusted to 200°C and that of quadrupole was set at 150°C. GC-MS was operated using electron impact ionization with a 60–600 atomic mass unit (amu) scan range. The initial temperature of column was kept at 70°C for 4 min. Then the temperature was ramped at 4°C /min to 220°C. Subsequently, the temperature was increased to 310°C by 8°C /min and was held for 10 min. Identification of the interested peaks was performed by searching the NIST database installed in the Thermo-Finnigan Trace DSQ GC-MS system and comparing with the peaks of commercial standards.

### Validation of GC-MS method

In order to validate the stability of the GC-MS system, an equal volume of each sample was pooled together to generate a pooled quality control (QC) sample [46], [47]. This QC sample was processed as real samples and then was randomly inserted amongst the real sample queue to be analyzed nine times accordingly. The system stability was expressed as the relative standard deviation (RSD) of the relative peak areas, i.e., the ratios of the peak areas of the metabolites to that of the internal standard. Eight common extracted ion chromatograms (EICs) shared by these injections were selected based on their relatively high abundance levels and wide retention time distribution range in the chromatogram. The result was 6.47%–12.63%, demonstrating the robustness of the method. This finding means that differences amid the test samples from different individuals were more likely to reflect varied metabolite profiles rather than analytical variation.

### Data processing

Data in instrument-specific format were converted to CDF format files. The program XCMS was used for nonlinear alignment of the data in the time domain and automatic integration and extraction of the peak intensities [48]. XCMS parameters were default settings except for the following: fwhm = 4, bw = 2 and snthersh = 3. The output data were imported into MATLAB software, where data were normalized using the summation of response of all metabolites in one sample and then calculated the ratios of the intensities of mass ions to that of the internal standard fragment ion (*m/z* 216.9, it is the most abundance fragment ion for the silylation derivative of ribitol).

It should be noted that GC-MS data inherently contain apparent variability and complexity such as multiple fragment ions from a single compound. Directly concatenating the matrices of processed-MS data is suboptimal as this may result in a matrix with an unfavorable samples-to-variables ratio [49]. It is necessary to use properly reduced matrix to conduct multivariate statistical analysis. A simple strategy was untargeted filtration of ion peaks using our in-house scripts in the MATLAB 7.0 (The MathWorks, Inc., USA), where the most abundant fragment ion with the same retention time (the time bin is 0.01 min) was remained and the other ions were excluded. By untargeted filtration of ion peaks, the data set was simplified and 170 ion peaks were obtained. The processed data list was further subjected to statistical analysis.

### Statistical analysis

Statistically significant differences in mean values were tested by one-way ANOVA and the Tukey post hoc test for comparisons of multiple groups. The differences were considered significant when *p*<0.05.

Prior to multivariate analysis, the data list was log-transformed and normalized. The principal component analysis (PCA) using SIMCA-P (version 11, Umetrics) was used to uncover unknown trends in the control, DOX and DOX plus *SND* groups based on myocardial metabolites. The Elastic Net using the library “glmnet” in the R environment (http://www.r-project.org) was used for classification and selection of significant ion peaks between control and DOX groups. The significant peak changes between samples were confirmed by manual quantification by calculating the area under the peak from raw chromatograms.

## Supporting Information

Table S1 **The relative levels and one-way ANOVA results of the potential biomarkers in the myocardial tissues of control, DOX and DOX plus** ***SND*** **group.**
(DOC)
