# Supplementary material for: Potential Biomarkers in Mouse Myocardium of Doxorubicin-Induced Cardiomyopathy: A Metabonomic Method and Its Application
Source: PLoS One. 2011 Nov 16;6(11):e27683. doi: 10.1371/journal.pone.0027683 (PMC3218026; doi:10.1371/journal.pone.0027683)
Supplement: Table S1 — The relative levels and one-way ANOVA results of the potential biomarkers in the myocardial tissues of control, DOX and DOX plus SND group. (DOC) [file pone.0027683.s001.doc]

Supplementary material

**Potential biomarkers in mouse myocardium of doxorubicin-induced cardiomyopathy: a metabonomic method and its application**

Guangguo Tan, Ziyang Lou, Wenting Liao, Zhenyu Zhu, Xin Dong, Wei Zhang, Wuhong Li, Yifeng Chai

Table s1. the relative levels and one-way ANOVA results of the potential biomarkers in the myocardial tissues of control, DOX and DOX plus *SND* group.

| No. | t_R_(min) | Metabolites | Control group (n=8) | DOX group (n=8) | DOX+*SND* group (n=8) | P value for ANOVA | |
| --- | --- | --- | --- | --- | --- | --- | --- |
|  |  |  |  |  |  | DOX &  CTR | DOX &  DOX+*SND* |
| 1 | 7.65 | Lactate | 7.6945 ± 0.4507 | 6.9495 ± 0.5177 | 7.5872 ± 0.4102 | 0.0108 | 0.0301 |
| 2 | 8.96 | L-Alanine | 1.1508 ± 0.0925 | 1.3942 ± 0.1593 | 1.1643 ± 0.1453 | 0.0047 | 0.0074 |
| 3 | 10.87 | β-Hydroxybutyric acid | 0.2864 ± 0.0455 | 0.2010 ± 0.0394 | 0.2478 ± 0.0470 | 0.0024 | 0.1091 |
| 4 | 12.58 | L-Valine | 0.2163 ± 0.0188 | 0.1697 ± 0.0326 | 0.2081 ± 0.0291 | 0.0073 | 0.0277 |
| 5 | 14.54 | Phosphate | 5.8897 ± 0.2967 | 6.8196 ± 0.4115 | 6.3673 ± 0.4881 | 0.0005 | 0.0899 |
| 6 | 15.15 | Isoleucine | 0.1156 ± 0.0089 | 0.0885 ± 0.0117 | 0.1066 ± 0.0176 | 0.0014 | 0.0324 |
| 7 | 15.48 | Glycine | 0.5616 ± 0.0756 | 0.7171 ± 0.0663 | 0.6065 ± 0.0806 | 0.0012 | 0.0190 |
| 8 | 16.21 | Succinate | 0.0026 ± 0.0006 | 0.0039 ± 0.0008 | 0.0031 ± 0.0005 | 0.0016 | 0.0412 |
| 9 | 18.09 | Threonine | 0.1436 ± 0.0227 | 0.1110 ± 0.0121 | 0.1199 ± 0.0164 | 0.0036 | 0.5796 |
| 10 | 21.47 | Malate | 1.7379 ± 0.1769 | 2.1891 ± 0.1879 | 1.8864 ± 0.2413 | 0.0007 | 0.0193 |
| 11 | 22.69 | Proline | 5.7699 ± 0.6164 | 6.8165 ± 0.7331 | 6.3511 ± 0.4543 | 0.0070 | 0.3017 |
| 12 | 23.40 | Threonic acid | 0.0155 ± 0.0028 | 0.0206 ± 0.0026 | 0.0188 ± 0.0010 | 0.0006 | 0.2726 |
| 13 | 25.36 | Glutamine | 0.2617 ± 0.0660 | 0.4168 ± 0.1000 | 0.3216 ± 0.0552 | 0.0015 | 0.0521 |
| 14 | 25.54 | Phenylalanine | 0.0277 ± 0.0043 | 0.0379 ± 0.0028 | 0.0321 ± 0.0058 | 0.0004 | 0.0437 |
| 15 | 28.85 | DHAP | 0.0008 ± 0.0002 | 0.0013 ± 0.0002 | 0.0011 ± 0.0002 | 0.0001 | 0.0566 |
| 16 | 29.13 | G-3-P | 0.1905 ± 0.0419 | 0.2611 ± 0.0470 | 0.2307 ± 0.0454 | 0.0129 | 0.3796 |
| 17 | 30.54 | Citrate | 0.0054 ± 0.0007 | 0.0036 ± 0.0006 | 0.0046 ± 0.0008 | 0.0002 | 0.0355 |
| 18 | 31.82 | Fructose | 0.0723 ± 0.0132 | 0.1004 ± 0.0217 | 0.0884 ± 0.0113 | 0.0059 | 0.3171 |
| 19 | 32.11 | Glucose | 2.5612 ± 0.4834 | 3.5706 ± 0.5931 | 2.9240 ± 0.4747 | 0.0024 | 0.0535 |
| 20 | 36.59 | Myo-Inositol | 0.6036 ± 0.1475 | 0.8589 ± 0.2099 | 0.7753 ± 0.0933 | 0.0105 | 0.5475 |
| 21 | 40.26 | Linoleic adid | 0.0250 ± 0.0062 | 0.0152 ± 0.0039 | 0.0193 ± 0.0022 | 0.0006 | 0.1754 |
| 22 | 40.94 | Stearic acid | 0.0429 ± 0.0089 | 0.0609 ± 0.0119 | 0.0487 ± 0.0069 | 0.0030 | 0.0460 |
| 23 | 44.14 | Arachidonic acid | 0.0029 ± 0.0007 | 0.0020 ± 0.0006 | 0.0026 ± 0.0003 | 0.0081 | 0.1039 |
| 24 | 51.34 | Cholesterol | 0.0539 ± 0.0150 | 0.0745 ± 0.0139 | 0.0706 ± 0.0087 | 0.0109 | 0.8187 |
